# Insecticide-treated nets provide protection against malaria to children in an area of insecticide resistance in Southern Benin

**DOI:** 10.1186/s12936-017-1873-1

**Published:** 2017-05-26

**Authors:** John Bradley, Aurore Ogouyèmi-Hounto, Sylvie Cornélie, Jacob Fassinou, Yolande Sissinto Savi de Tove, Adicath Adéola Adéothy, Filémon T. Tokponnon, Patrick Makoutode, Alioun Adechoubou, Thibaut Legba, Telesphore Houansou, Dorothée Kinde-Gazard, Martin C. Akogbeto, Achille Massougbodji, Tessa Bellamy Knox, Martin Donnelly, Immo Kleinschmidt

**Affiliations:** 1 MRC Tropical Epidemiology Group, London School of Hygiene and Tropical Medicine, Keppel Street, London, WC1E 7HT UK; 2 Unité d’Enseignement et de Recherche en Parasitologie-Mycologie de la Faculté des Sciences de la Santé de Cotonou, Cotonou, Benin; 3 Institut Régional de développement/Centre de Recherche Entomologique de Cotonou, Cotonou, Benin; 4 Programme National de Lutte contre le paludisme Cotonou, Cotonou, Benin; 5 World Health Organization, Geneva, Switzerland; 6 Liverpool School of Tropical Medicine, Liverpool, UK

**Keywords:** Malaria, Insecticide, Pyrethroid, Resistance, Nets

## Abstract

**Background:**

Malaria control is heavily reliant on insecticides, especially pyrethroids. Resistance of mosquitoes to insecticides may threaten the effectiveness of insecticide-based vector control and lead to a resurgence of malaria in Africa.

**Methods:**

In 21 villages in Southern Benin with high levels of insecticide resistance, the resistance status of local vectors was measured at the same time as the prevalence of malaria infection in resident children.

**Results:**

Children who used LLINs had lower levels of malaria infection [odds ratio = 0.76 (95% CI 0.59, 0.98, p = 0.033)]. There was no evidence that the effectiveness of nets was different in high and low resistance locations (p = 0.513). There was no association between village level resistance and village level malaria prevalence (p = 0.999).

**Conclusions:**

LLINs continue to offer individual protection against malaria infection in an area of high resistance. Insecticide resistance is not a reason to stop efforts to increase coverage of LLINs in Africa.

## Background

Deaths caused by malaria have fallen from an estimated 839,000 in 2000 to 438,000 in 2015 [1]. Much of this decline is due to insecticides: in 2014, 59% of the sub-Saharan African population were protected by either long-lasting insecticidal nets (LLINs) or indoor residual spraying (IRS), compared to 2% in 2000 [1]. If LLINs and IRS ceased to be effective, many of the public health gains could be reversed. Unfortunately, this is a possibility since resistance to insecticides in *Anopheles* malaria vectors is now widespread [2]. This is especially true for insecticides in the pyrethroid class, the sole class of insecticide used in LLINs [2].

Although there is extensive entomological evidence that pyrethroids are becoming less effective at killing mosquitoes [2], the impact on epidemiological outcomes is not clear. Examples of apparent malaria control failure due to pyrethroid resistance are not conclusive nor generalizable. For example, a steep decline in malaria incidence in South Africa after DDT replaced pyrethroids for IRS often cited as an example of control failure due to resistance could also be due to a concurrent introduction of artemether/lumefantrine for malaria treatment [3]. There is also some evidence of pyrethroid IRS failing on Bioko Island, Equatorial Guinea [4–6], but again this is not conclusive. There are no convincing examples of control failure due to resistance in areas where LLINs are the primary form of malaria control. Studies in Malawi and Kenya have shown that LLINs continue to provide protection [7, 8]. Those studies examined the impact of resistance on infection incidence. This study looks at the impact of resistance on prevalence. Because of the lack of evidence on this important issue, a study to measure the impact of insecticide resistance on epidemiological outcomes was launched in five countries; this paper reports on the results of the assessment conducted in Benin, West Africa [9]. In Southern Benin resistance to pyrethroids is high [10] and LLINs are the primary form of vector control, with two pyrethroid-treated nets (deltamethrin and permethrin) in widespread use. In a sample of 21 villages, standard WHO susceptibility tests [11] were used to measure mortality of mosquitoes exposed to these insecticides; and this was compared to prevalence of malaria infection in children.

The study addressed two questions:

1. Does resistance to pyrethroids reduce the effectiveness of LLINs for malaria prevention?

2. Are higher levels of resistance to pyrethroids associated with a greater prevalence of malaria?

## Methods

The study was conducted in 21 villages (subsequently referred to as clusters) in four rural districts in the Plateau Department of Benin: Ifangni, Sakete, Ketou and Pobe. The total area is 3264 km^2^ and the population is approximately 400,000. There are two rainy seasons: April–July and September–October. The main malaria vectors are *Anopheles gambiae* s.s. and *Anopheles coluzzii*; both species are found in most clusters [12]. In West Africa, there is extensive introgression of resistance mutations between these species [13] and in Benin in particular levels of resistance are very similar and, therefore, they are treated as a single entity here [14]. The National Malaria Control Programme has distributed LLINs treated with deltamethrin (PermaNet^®^ 2.0 [Vestergaard] and DawaPlus^®^ 2.0 [Tana Netting]) and permethrin (Olyset^®^ Net [Sumitomo]) in the Plateau Department.

Mosquito larvae were collected from breeding habitats once in each cluster. The sampling took place between June and August 2015. Whenever possible, more than 6 larval habitats were examined in each cluster. Larvae were reared in an insectary with relative humidity 80 ± 10% and temperature 25 ± 2 °C. Adult mosquitoes were maintained with 10% honey water after emergence. Bioassays were performed on 2–5 day old females using either deltamethrin or permethrin at the standard WHO diagnostic concentrations of 0.05 and 0.75% respectively [11]. The laboratory susceptible reference strain of *An. gambiae* (Kisumu) was used to check the quality of the impregnated paper. One hour after exposure, mosquitoes were transferred to holding tubes and fed with 10% honey water. Mortality was recorded 24 h post-exposure. Additional mosquitoes were exposed to untreated papers to assess control mortality.

Cross sectional surveys were carried out in each cluster in July 2015. In each cluster forty households were randomly selected from a list (which came from census carried out in 2013). Each household was visited, and if anyone was present, written informed consent was sought to participate in the study. An adult was asked questions about socio economic indicators and bed net use. Up to 3 children per household aged between 6 months and 10 years were chosen at random for malaria microscopy. Blood slides were read by 2 microscopists and if they disagreed on the presence of malaria parasites, a third reader adjudicated. Those testing positive were traced and treated according to national guidelines or referred to a local health facility.

Logistic regression was used to calculate odds ratios (ORs) for the effect of net use on malaria infection prevalence; the linear effect of a 10% increase in mosquito mortality on infection prevalence; and whether prevalence was different in higher and lower resistance clusters (defined in terms of the median mortality). The effect of nets was calculated separately in high and low resistance clusters; an interaction parameter in the model was used to assess if the effect of nets differed by resistance status. The sample size of 21 clusters was based on having 80% power to detect an increase from 20% prevalence [10] in the 11 lower resistance clusters to 30% in the 10 higher resistance clusters, with a sample of 80 children per cluster and a coefficient of variation of 0.25. Estimates adjusted for socio-economic status (SES), age and net use were also calculated. SES was calculated using principal component analysis and divided into quartiles. Random effects for clusters were used to account for intra cluster correlation in responses. The Spearman correlation coefficient was used to assess the association between cluster level permethrin and deltamethrin mortalities.

This study was approved by the Benin Ministry of Health, the National Ethics Committee for Health Research at the Ministry of Health. Written consent was obtained from all participants, who were informed of objectives of the study and the advantages and disadvantages of participation.

## Results

Mortality of mosquitoes exposed to deltamethrin was measured in all 21 clusters. The median number of mosquitoes exposed per cluster was 81 [inter quartile range (IQR) 53–101]. Median mosquito mortality was 55.2% (IQR 47.4–68.5%). Mortality to permethrin was measured in 20 of the 21 clusters. The median number of mosquitoes exposed was 25 (IQR 22–35). Median mortality was 18.2% (IQR 8.1–32.2%). In all assays there was 0% mortality in the control group of mosquitoes. There was poor correlation between cluster specific deltamethrin and permethrin mortality (correlation coefficient = 0.20, p = 0.407) and therefore analyses were conducted separately for each active ingredient.

In the 21 study clusters, 1621 children from 813 households had blood taken for microscopy. Of these 836 (51.6%) had a malaria infection. Nets were used by 1231 (75.9%) of the children the night before the survey. Net use was associated with lower risk of malaria infection, OR 0.76 (95% CI 0.59, 0.98, p = 0.033) (Table 1). Prevalence was not significantly different in children who used a deltamethrin compared to a permethrin net: OR 1.13 (95% CI 0.76, 1.69, p = 0.543).

**Table 1:** Effectiveness of nets

Area	Reported use of net the previous night	Malaria prevalence in children aged 6 months to 10 years, % (n/N)	Odds ratio (95% CI)	Adjusted odds ratio^a^ (95% CI)
All clusters	No	54.1% (211/390)	1	1
	Yes	50.8% (625/1231)	0.76 (0.59, 0.98), p = 0.033	0.77 (0.60, 1.00), p = 0.053
Lower resistance to deltamethrin (mosquito mortality >55.2%)	No	52.2% (107/205)	1	1
	Yes	49.6% (306/617)	0.82 (0.58, 1.17), p = 0.282	0.85 (0.59, 1.22), p = 0.381
Higher resistance to deltamethrin (mosquito mortality <55.2%)	No	56.2% (104/185)	1	1
	Yes	52.0% (319/614)	0.69 (0.48, 1.00), p = 0.052	0.70 (0.48, 1.02), p = 0.061
	Interaction parameter for the difference in net effectiveness between higher and lower resistance clusters		0.84 (0.51, 1.41) p = 0.513	0.82 (0.49, 1.38) p = 0.457
Lower resistance to permethrin (mosquito mortality >18.2%)	No	60.1% (113/118)	1	1
	Yes	51.4% (304/592)	0.73 (0.51, 1.04), p = 0.082	0.75 (0.52, 1.07), p = 0.115
Higher resistance to permethrin (mosquito mortality <18.2%)	No	49.5% (95/196)	1	1
	Yes	51.6% (292/566)	0.84 (0.58, 1.20), p = 0.335	0.86 (0.59, 1.24), p = 0.416
	Interaction parameter for the difference in net effectiveness between higher and lower resistance clusters		1.15 (0.69, 1.92) p = 0.595	1.15 (0.68. 1.94) p = 0.600

^a^Adjusted for age and SES

There was no evidence that the effect of nets was different in clusters with lower [OR 0.82 (95% CI 0.59, 1.17), p = 0.282] or higher [OR 0.69 (95% CI 0.48, 1.00), p = 0.052] resistance to deltamethrin (interaction p = 0.513) (Table 1) or for lower [OR 0.73 (95% CI 0.51, 1.04), p = 0.082] or higher [OR 0.84 (95% CI 0.58, 1.20), p = 0.335] resistance to permethrin (test for interaction: p = 0.595).

Malaria prevalence was similar in clusters with lower resistance to deltamethrin compared to those with higher resistance OR 1.11 (95% CI 0.65, 1.90, p = 0.698) (Table 2); this was true even when restricted just to those children who slept under deltamethrin treated nets OR 0.82 (95% CI 0.44, 1.53, p = 0.532). The estimated effect of a 10% increase in mosquito mortality was negligible: OR 1.00 (95% CI 0.85, 1.17, p = 0.999). There was also little association between malaria prevalence in clusters with lower resistance to permethrin compared to clusters with higher resistance OR 0.88 (95% CI 0.50, 1.53, p = 0.640) (Table 2), even when restricted to children who slept under permethrin treated nets OR 0.66 (95% CI 0.28, 1.54, p = 0.336). The estimated effect of a 10% increase in mosquito mortality was negligible: OR 0.99 (95% CI 0.90, 1.09, p = 0.893).

**Table 2:** The association between deltamethrin and permethrin mortality on malaria prevalence

Insecticide	Effect of		Malaria prevalence in children aged 6 months to 10 years, % (n/N)	Odds ratio (95% CI)	Adjusted odds ratio^a^ (95% CI)
Deltamethrin	Dichotomized resistance	Lower resistance (mosquito mortality >55.2%)	50.2% (413/822)	1	1
		Higher resistance (mosquito mortality <55.2%)	52.9% (423/799)	1.11 (0.65, 1.90), p = 0.698	1.04 (0.60, 1.81), p = 0.877
	Linear increase in mosquito mortality	10% increase	–	1.00 (0.85, 1.17), p = 0.999	1.03 (0.88, 1.21), p 0.734
Permethrin	Dichotomized resistance	Lower resistance (mosquito mortality >18.2%)	53.5% (417/780)	1	1
		Higher resistance (mosquito mortality <18.2%)	50.8% (387/762)	0.88 (0.50, 1.53), p = 0.640	0.86 (0.49, 1.51), p = 0.600
	Linear increase in mosquito mortality	10% increase	–	0.99 (0.90, 1.09), p = 0.893	0.99 (0.90, 1.08), p = 0.765

^a^Adjusted for age, SES and net use

## Discussion

While insecticide resistance in malaria vectors is widespread [2], there is little evidence of its impact on the effectiveness of nets and how in turn this influences epidemiological outcomes [2, 9, 15]. This study found that use of insecticide treated nets was associated with lower risk of malaria infection in an area of high pyrethroid resistance. Furthermore, there was no evidence that the effect of nets differed in villages stratified by vector resistance. The study also found no association between pyrethroid resistance and malaria prevalence. The fact that no impact of pyrethroid resistance on net effectiveness and malaria prevalence was detected in this study is both an interesting and comforting result. It suggests that whilst insecticide resistance is a grave threat to the long-term sustainability of malaria control it has not apparently reached a level, at least in Benin, where it would render LLINs ineffective. Control programmes should continue to strive to attain high LLIN coverage at the same time researchers and industry seek to develop alternative control options.

Nets present a physical barrier to the mosquito as well as the protection offered by the repellent and killing effect of insecticide. In addition to the physical barrier, a recent meta-analysis found that insecticide treated nets still offered greater protection than untreated nets when local vectors are resistant to pyrethroids [16]. Furthermore, even if insecticides no longer kill mosquitoes, there could still be an excito-repellent effect. There are, therefore, plausible mechanisms through which LLINs continue to provide personal protection in the face of pyrethroid resistance.

On top of personal protection offered by LLINs, if the coverage of nets is high enough there is a mass effect [17] which benefits both users and non-users by reducing the lifespan of mosquitoes. If resistance attenuated the mass effect of LLINs without affecting personal protection, one would expect to see a greater difference in malaria risk between users and non-users in areas of high resistance than in areas of low resistance; a difference which could be further exacerbated by diverting mosquitoes from users to non-users without killing them; no such difference was observed in this study.

It would, however, be reckless to conclude from this study that insecticide resistance has no impact on malaria transmission. There are a number of reasons why an association between resistance and malaria prevalence might not have been observed in this study, even if there is an impact of resistance on malaria control. Resistance to pyrethroids was observed in all study clusters [11] and, therefore, there may be an impact of resistance on malaria prevalence across all study clusters but the absence of truly susceptible mosquito populations and a relatively insensitive resistance definition prevents us detecting an effect.

While mortality 24 h post exposure is a pragmatic test for the presence of resistance, it does not necessarily mean it is a good measure of the strength of resistance in resistant mosquito populations. Moreover, recent data suggests that even in mosquitoes classified as resistant by discriminant dose tests there are likely to be epidemiologically important sub-lethal effects [18]. Molecular screening of resistance mechanisms or measures of resistance based on a dose response relationship may be a more informative method of characterization although only the former is likely to be able to be pushed to the scale that studies of this nature require [19].

Since it is not possible to randomize villages to different levels of insecticide resistance and not ethical to randomize children to not using nets, all studies of this issue must be observational—and, therefore, subject to confounding. In this study, two important confounders (age and SES) were adjusted for which made little impact on the analysis, but some residual confounding may remain [9].

Nonetheless, this study found that LLINs continued to offer individual protection in an area of high insecticide resistance and found no association between resistance and malaria prevalence where LLIN use was high. Insecticide resistance threatens the major gains that have been made in reducing malaria disease burden, but whilst for alternative control approaches are searched for efforts should be redoubled on to increase access to LLINs in Africa.
